# Using Virtual Environments to Improve Real-World Motor Skills in Sports: A Systematic Review

**DOI:** 10.3389/fpsyg.2019.02159

**Published:** 2019-09-20

**Authors:** Stefan C. Michalski, Ancret Szpak, Tobias Loetscher

**Affiliations:** Cognitive Ageing and Impairment Neurosciences Laboratory, School of Psychology, University of South Australia, Adelaide, SA, Australia

**Keywords:** virtual environment, transfer, study design, training environment, learner characteristics

## Abstract

In many settings, sports training can be difficult to organize, logistically complicated and very costly. Virtual environments (VE) have garnered interest as a tool to train real-world sports skills due to the realism and flexibility that they can deliver. A key assumption of VE-based training is that the learned skills and experiences transfer to the real world, but do they? Using PRISMA guidelines, this systematic review evaluated the available evidence regarding the transfer of motor skills from VE training to real-world sporting contexts. The initial search identified 448 articles, but only 4 of these articles met basic criteria necessary to assess real-world transfer. Key factors regarding the study design, learner characteristics and training environment of these studies are considered. In a relatively new area of research, the findings from these 4 articles are encouraging and provide initial support for the notion that skills training in a VE can improve real-world performance in sports. However, for a wider uptake of VEs in sports training, it is important that more research demonstrates real-world transfer. Study design recommendations are suggested for researchers, developers or trainers who are considering demonstrating real-world transfers from virtual to real-world environments.

## Introduction

Practice makes perfect. Perhaps there is some truth in this popular phrase, as mastering any skill, be it performing a backflip, playing the guitar or serving a tennis ball requires practice. But what makes practice perfect? Despite over a century of research, it is still a matter of debate how training should be structured to maximize the potential for learning (Guadagnoli and Lee, 2004). What is clear is that to attain expertise in a sport, athletes must devote a considerable amount of time to training (Miles et al., 2012). Yet, often training can be difficult to organize, logistically complicated and very costly. Consider a quarterback who needs to pinpoint a pass amongst an entire field of players, a skier who needs access to a snow-covered mountain and a race car driver who needs access to a vehicle on a vacant race track. To overcome these barriers, heavy demands have been placed on finding contemporary, cost-efficient and flexible training methods (Gupta et al., 2008). Virtual environments (VE) have garnered interest as a tool for training real-world skills due to the realism and flexibility that they can deliver.

As technology is rapidly improving and becoming more affordable, many fields and professions have started using VEs as a tool to train real-world skills. A variety of professions such as surgeons (Seymour et al., 2002), pilots (Hays et al., 1992) and firefighters (Stansfield et al., 2000), to name a few, have been shown to benefit from training in a VE. Due to the inherent level of risk associated with such training programs, which typically involve “learning by doing,” there is a growing trend in shifting toward VE-based training programs (Gavish et al., 2015). VEs promise a safe, realistic and interactive learning environment with the opportunity for repeated practice, supported by feedback and standards to measure performance. However, there is still a long way to go and further technological advances need to be made so that VEs can reach their full potential.

While VEs are becoming increasingly popular in fields such as aviation and surgery, its use in sports training is still rather limited. Yet, there are many potential advantages to VE-based training in a sporting context. VEs can be used to simulate the presence of team members and opponents, allow coaches to create personalized scenarios for players (Kim et al., 2013; Düking et al., 2018), practice can be designed relative to the skill level of the performer (Düking et al., 2018), users can log their performance and closely monitor their development (Neumann et al., 2018) and numerous sporting environments can be simulated. With the added benefit of being able to train in a safe and repeatable environment, VEs appear to be a promising platform to improve real-world motor skills in sports.

While promising, it is important to point out that VE training is not useful or practical for training in all sports. For example, water sports such as swimming cannot be trained in VEs. The current state of technology makes it also challenging to simulate training for skills relying on highly accurate haptic feedback and multiplayer interactions. The technical limitations and the costs associated with the creation of virtual training environments pose a significant barrier for an uptake in VE sports training. Overviews of technical requirements for VEs can be found in Miles et al. (2012) and Petri et al. (2018). However, it is important to note that even if some sports are currently too challenging and costly to simulate with high fidelity, VEs might be still useful for observational learning (Tanaka, 2017). Users can potentially improve their skills by passively viewing a skill demonstration or instructional video in which they can feel immersed.

An important prerequisite for a wider uptake of VE sports applications are demonstrations that the training leads to better performance in the real-world sport (Lathan et al., 2002; Neumann et al., 2018). That is, the trained skills can transfer to the real-world setting. Transfer has been defined as the process by which skills, abilities and knowledge developed through training are applied in a real-world situation or task (Baldwin and Ford, 1988). Burke and Hutchins (2007) propose three important factors to consider when evaluating transfer of training, including study design; learner characteristics; and training environment. Each of these factors will be addressed in turn.

An adequate study design is essential when evaluating transfer of training (Gray, 2017). Firstly, to determine the effectiveness of VE-based training, researchers must include a group to control for basic practice effects (Abernethy and Wood, 2001). Secondly, it is insufficient to assess the effectiveness of VE training by only quantifying the extent of improvement on the VE training task (near transfer) (Gray, 2017). This is because the results can almost always be expected to be positive, mainly due to practice effects. Thus, studies need to include an assessment of performance from the training task to the real-world sport (far transfer) (Abernethy and Wood, 2001; Gray, 2017).

Characteristics of the learner are important to consider when evaluating the effectiveness of training (Baldwin and Ford, 1988). Examples of characteristics which have been identified as affecting transfer of training include cognitive ability, motivation, personality and prior level of experience in the task (Sackett et al., 1998). One aspect to consider in relation to sports training is the user's prior level of experience in the sport being assessed. Guadagnoli and Lee (2004) suggest that training is optimal when the difficulty of the task is matched to the skill level of the individual. Adaptive training is based on this theory whereby training is appropriately matched to the user's level of success in a training bout. Adaptive training is when the difficulty of the task is constantly adjusted (usually by systematically increasing the difficulty) to ensure the training is always challenging and engaging. Perhaps the effectiveness of VE-based training does increase when the training is challenging (relative to the skill level of the individual).

The third factor identified which is necessary to consider when evaluating transfer is the training environment. The most compelling VEs give users a subjective experience of presence and immersion by engaging multiple sensory modalities, providing both a realistic and engaging experience in the training environment (Witmer and Singer, 1998). Presence refers to the subjective experience of “being there” in a VE, while immersion refers to the technological capabilities of delivering this experience (Witmer and Singer, 1998). Presence and immersion are highly related to one another, as they are both necessary to convey a realistic experience to the user (Slater and Wilbur, 1997). Based on advancements in VE technology in recent times, presence and immersion have become much easier to create (Fox et al., 2009). VEs are hypothesized to be better training tools in comparison to standard computer-based training programs and training using video because of the greater level of realism they can offer (Witmer and Singer, 1998). Perhaps, transfer of training is dependent upon the capabilities of the VE to create feelings of presence and immersion within the user (Vignais et al., 2015).

To maximize the effectiveness of VE training, the physical and cognitive fidelity of the training environment should also be considered. Hochmitz and Yuviler-Gavish (2011) proposed two complementary aspects. Firstly, it is assumed that for a positive transfer to occur, VEs must replicate high physical fidelity regarding the real-world environment. This involves the degree to which the VE looks, feels (via haptic rendering) and sounds (via auditory rendering) like the real-world situation (Alexander et al., 2005). Secondly, it has also been proposed that the VEs must replicate high cognitive fidelity regarding the real-world environment. This involves the degree to which VEs can engage users in the types of cognitive activities (i.e., stimulus-response relationship), such as the playing strategy and decision-making that is involved in the real-world task (Lathan et al., 2002). For example, a player in a competitive game of table tennis requires concentration (stimulus) and quick decision making (response). The underlying level of physical fidelity and cognitive fidelity should be evaluated as a potential factor affecting outcomes.

An additional factor that might influence the effectiveness of transfer are the characteristics of the sport trained. Broadly speaking, sports can be categorized into open and closed skills. Closed skill sports are defined as sports that involve a predictable, consistent and self-paced environment (e.g., cycling, golf, skiing) (Wang et al., 2013). Contrastingly, open skill sports are defined as sports that require players to engage in an unpredictable, constantly changing and externally-paced environment (e.g., soccer, cricket, rugby) (Wang et al., 2013). For example, having opponents or teammates can change the pace and predictability of sport interactions. Hence, a player must rely on open skills and their ability to quickly adapt to external changes in the competitive sport environment. Wang et al. (2013) suggest that open skill sports typically require athletes to exhibit greater flexibility in visual attention, decision making and action execution. An interesting question is thus whether the effectiveness of VE training differs between open and closed skills sports.

The aim of this review is to synthesize the evidence for the effectiveness of virtual environments as a tool to train real-world motor skills in sports. Although VEs offer potential as a tool for sports training, it is necessary to first establish whether VEs are an effective tool to improve real-world skills by reviewing articles that demonstrate real-world transfer. Factors related to the study design, learner's characteristics and training environment have been proposed to impact the transfer of trained skills. An investigation of these factors can add to both theoretical and practical knowledge to maximize the effectiveness of training in VEs for improving real-world sport skills.

## Methods

### Search Strategy

The preferred reporting items for systematic reviews and meta-analyses (PRISMA) guidelines were followed throughout the review process (Liberati et al., 2009). A literature search was performed on February 16, 2019, using the following databases: PsycINFO, SportDiscus and IEEE Explore. For all mentioned databases, the following search was conducted: [(“virtual reality” OR “virtual environment”) AND (sport^*^ OR “motor skill^*^”) AND (transfer OR learn^*^ OR train^*^)]. Psychology, sports and engineering databases were used to cover the scope of this review, regarding transfer, sport and virtual technology.

### Inclusion and Exclusion Criteria

Each article in this review must have included a VE and an assessment of real-world sports performance before and after VE training.

To have been considered a VE in this review, it must have included all the following components: (i) display or projection of an image e.g., head-mounted display (HMD) or Cave Automatic Virtual Environment (CAVE) or Powerwall; (ii) interactivity within the environment is essential via tracking of the user's movements; (iii) provision of sensory feedback (e.g., visual, auditory or haptic); (iv) software to render three-dimensional depth cues (Gray, 2017).

The definition by Oxford Dictionary of Sports Science and Medicine for sport was used in this review, “an activity involving physical exertion and skill in which an individual or team competes against another or others” (Kent, 2006).

An assessment of transfer must have included a group to control for basic practice effects and a measure of far transfer to assess the effect on real-world performance. These are the most basic elements necessary to assess transfer.

Only articles published in English were included, inclusive of articles from any year of publication. Each study must have included a healthy population. Articles were excluded in the review if a child or clinical sample were used and if it were not an original peer-reviewed research paper, such as a conference paper, dissertation or review.

### Article Selection

**Figure 1:**
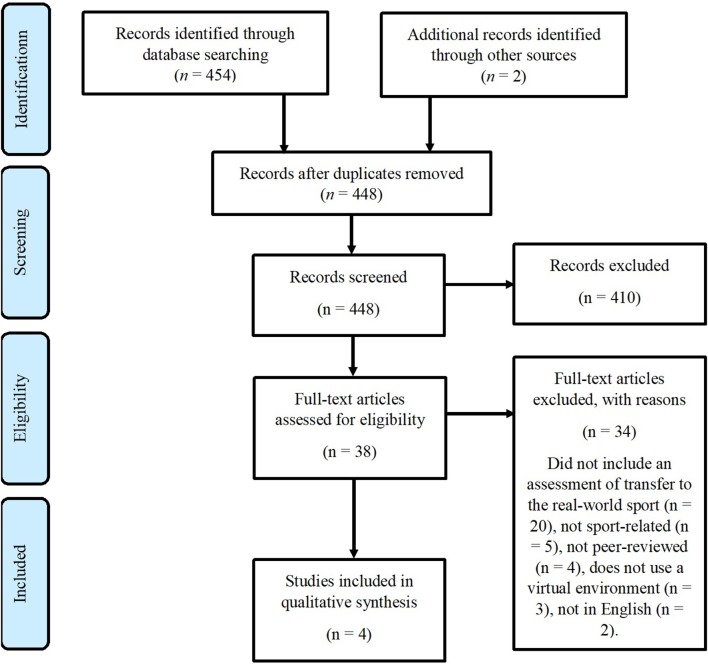
The article selection and screening process using the PRISMA flow diagram (Liberati et al., 2009). Databases searched included PsycINFO, SportDiscus via EBSCOhost and IEEE Explore.

Two reviewers completed the article selection and screening process in this review. Covidence (2018) was used throughout the screening process to manage articles. Titles and abstracts were screened to identify studies that appeared eligible for inclusion. Full-text articles were sourced and read for articles that appeared eligible, or for which eligibility could not be determined. During full-text reading, articles were either included in the review or excluded with reasons based on the criteria, listed in Figure 1. Additionally, reference lists of included articles were scanned for additional articles and entered the start of the review process. If a conflict arose during any stage during the article selection and screening process, the reviewers resolved the dispute via discussion; until consensus was reached.

### Data Extraction

Data extracted from selected studies included: major findings, participant demographics, factors regarding the study design, learner characteristics and training environment. To determine the level of physical and cognitive fidelity in each study the two reviewers reached a consensus based on the information provided in the article.

## Results and Discussion

A total of 456 articles were selected for initial screening according to PRISMA protocol (Liberati et al., 2009). After removal of eight duplicates, 448 articles remained for screening. After screening titles and abstracts, 38 articles met inclusion criteria and were selected for review. Thirty four articles were excluded with reasons provided in Figure 1. In total four articles were included in this systematic review. A summary of the key characteristics and relevant findings of each of the included articles is included in Table 1.

**Table 1:** Summary of included articles.

**Authors**	**Gray (2017)**	**Rauter et al. (2013)**	**Tirp et al. (2015)**	**Todorov et al. (****1997****)**	
				**Experiment 1**	**Experiment 2**
Participants	*n* = 80	*n* = 8	*n* = 38	*n* = 42	*n* = 21
	Age: 17–18	Age: 28–45	Age: (*M* = 25.2)	Age: N/A	Age: N/A
	Sex: Male = 80, Female = 0	Sex: Male = 4, Female = 4	Sex: Male = 26, Female = 12	Sex: N/A	Sex: N/A
Sport	Baseball batting	Rowing	Dart throwing	Table Tennis	Table Tennis
Level of experience in the sport being assessed	Intermediate. Participants were baseball players who played competitive high school baseball in the United States at the time of training	Intermediate. Participants were recreational rowers without competition experience and complete <2 h of training per week	Novices. All participants were inexperienced in dart throwing	Novices. Information regarding participants table tennis experience was not specified	Novices. Information regarding participants table tennis experience was not specified
Task in VE training	Hit virtual baseball with a real baseball bat	Training co-ordination of body movement and handling oars	Throwing darts at a bullseye	Returning shots and hitting targets	Returning shots and hitting targets
Measure of real-world sports performance for pre- and post-test	Hitting real baseballs via pitching machine	Quantitative biomechanical performance measures and qualitative video evaluation	15 throws at a dart board	50 attempts to hit targets on a real-world table tennis table	50 attempts to hit targets on a real-world table tennis table
Virtual technology used in the study	LCD screen placed in front of the participant. The VE projected an incoming baseball, pitcher and the playing field	A custom-built rowing machine was placed in a CAVE display	A dartboard was projected on the wall and Xbox Kinect sensors were used to track participant's actions	Participants stood next to a computer screen that simulated a table tennis table, moving paddle and ball	Participants stood next to a computer screen that simulated a table tennis table, moving paddle and ball
Length of VE intervention	9 h	3 h, 20 min	Did not report	10 min	Did not report
Research Designs	Experimental (between-subjects)	Experimental (between-subjects)	Experimental (between-subjects)	Experimental (between-subjects)	Experimental (between-subjects)
Groups in study	1) Adaptive training in VE; 2) Extra batting sessions in VE; 3) Extra batting in real-world; 4) No training.	1) VE training ;2) Real-world training (on open water).	1) VE training; 2) Real-world training; 3) No training.	1) VE training ;2) Real-world training (coaching).	1) VE training; 2) Real-world training (extra practice).
Real-world sports performance significantly improved pre-post virtual training	Yes	No	Yes	Yes	Yes
VE training group significantly improved in comparison to a control group receiving no training.	Yes	N/A	Yes	N/A	N/A
VE training group significantly improved in comparison to a control group receiving real-world training.	Yes	No	No	Yes	Yes
Physical fidelity	High	Moderate	Low	Low	Low
Cognitive fidelity	Moderate	Low	Moderate	Low	Low
Open or closed skill training	Closed	Closed	Closed	Closed	Closed
Adaptive training	Yes	No	No	No	No

*M, Mean; SD, Standard Deviation; N/A, Not available*.

### Description of Studies

Of the four articles included in this review, one reported multiple experiments (Todorov et al., 1997), resulting in a total of five studies included in the review. Out of the five studies there is a combined total of 189 participants. The earliest studies were published in 1997 (Todorov et al., 1997), while the remaining studies were published between 2013 and 2017. In the five studies included in this review, sample sizes ranged from 8 to 80. The length of the VE intervention varied between studies. The longest time spent training in a VE was reported as a total of 9 h (Gray, 2017), while the shortest time reported was 10 min (Experiment 1) (Todorov et al., 1997). Two studies did not specify the length of VE intervention.

While there are only four articles included in this review, there is an abundance of research assessing sports training in a VE (Bideau et al., 2003; Croft et al., 2011; Zaal and Bootsma, 2011; Marchal-Crespo et al., 2013; Miles et al., 2013), although these studies demonstrate an improvement in the VE-based sport task, the researchers do not assess the impact that the training had on real-world performance. A measure of real-world performance is essential in determining the benefit and value of the training. Notably, 20 out of 34 studies were excluded during full-text screening as they did not include a measure of far transfer. These elements are the minimum requirements to assess VE transfer in sports, yet only five studies in four articles met this search criteria.

Out of the five studies included in this review, a total of four different sports have been assessed, including darts (Tirp et al., 2015), rowing (Rauter et al., 2013), baseball (Gray, 2017) and table tennis (Todorov et al., 1997). In this review, all sports were eligible for inclusion. Thus, it may surprise that only four different sports assessed far transfer. A key reason for the limited number of sports might be that with the current state of technology, VEs are not ideal training platforms for all sporting interactions, particularly for in-water and interactive multi-player activities. The high costs for creating VEs with high levels of realism combined with uncertain VE training benefits may have been another reason for a slow uptake of VEs across sports. It is noteworthy, however, that the included studies comprised rather distinct simulations (e.g., darts and rowing). Although many of the sports in this review were comparably different, important comparisons regarding the study design, learner characteristics and training environment can be made.

### Study Design

The findings from studies assessing real-world performance improvements after VE-based training from pre-test to post-test, compared to no-training and compared to real-world training are considered in turn. Four out of five of the studies included in this review found that VE-based training led to a significant improvement in real-world performance from pre-test to post-test, as illustrated in Table 1. Though, considered by Abernethy and Wood (2001), to effectively assess transfer of training, studies need to include controls to rule out basic practice effects.

Two out of two studies found that training in a VE led to a significant improvement in real-world performance in comparison to a control group receiving no-training (Tirp et al., 2015; Gray, 2017). All available (however, limited) evidence suggests that VE-based training can enhance real-world performance compared to no training. This supports the growing interest in using VEs as a tool for training, especially as a complementary tool for when training in the real world is logistically difficult, dangerous or impractical to organize. While all current findings suggest that VE-based training improved real-world performance relative to no-training, it was found in three out of five studies that the amount of real-world improvement was significantly greater following VE training as compared to real-world training. Whether these findings are positively skewed due to a file drawer problem cannot be answered in this review.

Assessing transfer in a sport can be challenging when there are various aspects of sports performance to consider. Our results support the notion that motor skills can be improved after VE training. Although positive transfer was found in most studies, often the assessments utilized were narrow, involving trivial performance tasks to assess real-world performance. Todorov et al. (1997) found mixed effects in their study, as while target accuracy improved after VE training it was also found that their technique degraded. This highlights the importance of obtaining a comprehensive assessment of sports performance which future studies could consider to understand the true impact of the VE training.

Gray (2017) was the only study included in this review that assessed the impact of VE-based training in a competitive setting. In this study, 80 competitive baseball players were used (Gray, 2017). Each participant's league batting statistics (as assessed by on-base percentage) for the season following the training and their level of competition reached at a 5-year follow up was assessed. After training in a VE, participant's real-world batting performance and level of competition reached at the 5-year follow up were significantly higher when compared to groups that received no training and real-world training (Gray, 2017). These findings demonstrate the positive real-world implications that VE-based training can have in terms of athletic development and achievement in real competition with athletes of an intermediate level of experience. More comprehensive research is required, analyzing changes in real-world competitive situations which would provide further insight into the benefits that VE training can have on performance.

### Learner Characteristics

Sackett et al. (1998) proposed that learner characteristics affect transfer outcomes. For example, it has been found by numerous researchers that people with higher cognitive ability are better able to process and retain information in training (Colquitt et al., 2000; Velada et al., 2007; Grossman and Salas, 2011). However, none of the studies included in this review directly assessed learner characteristics such as cognitive ability, motivation and personality.

Two studies included participants with an intermediate level of experience in the sport being assessed (Rauter et al., 2013; Gray, 2017), while the remaining studies assessed novices. The skill level of the individual and the difficulty of the task in training are essential in the promotion of skill learning (Guadagnoli and Lee, 2004). Termed the challenge point framework, Guadagnoli and Lee (2004) propose that training is optimal when the level of challenge is relative to the skill level of the performer. Adaptive training is based on this concept, where the level of difficulty is suitably matched to the individual's level of success during training.

Gray (2017) investigated transfer of training from virtual to real-world baseball batting in athletes with an intermediate level of experience. A group that received adaptive training in a VE was compared to groups that received repetitive batting practice which involved hitting balls of the same speed and trajectory as released by a pitching machine (irrespective of the user's skill level) in both the real world and in a VE. In the VE adaptive training group, factors such as pitch speed and spin would regularly increase based on success or alternatively, decrease based on failure. Gray (2017) found that when training was adaptive (as constantly adjusted to the performer's skill level), it resulted in significantly greater improvements in real-world performance as compared to both groups that received the repetitive practice (in both the virtual environment and in the real world).

VE training has found to be most beneficial when taking advantage of the simulation to devise methods of training (i.e., adaptive training) which are difficult to implement in the real world. Gray (2017) attributed the improvement in the VE adaptive training group to the combination of ball types (i.e., speed, spin and trajectory) which is more realistic to the range of conditions players face in real-world gameplay. Perhaps, the added value is in being able to take advantage of the flexibility of VEs, rather than simply trying to recreate training in the real world. However, Gray (2017) is the only study included in this review to have assessed the effect of adaptive training as compared to other forms of training and more research comparing training forms are needed.

### Training Environment

The degree of physical fidelity and cognitive fidelity in the VEs varied considerably in each of the included studies. It might seem that the more similar the VE is to the real world the better the transfer of training (Miles et al., 2012). Yet, this review found that even simple displays and tasks were found to be beneficial, suggesting that perhaps a high degree of detail is not necessarily crucial to the success of skill acquisition. While Hochmitz and Yuviler-Gavish (2011) proposed that a high level of cognitive and physical fidelity is essential to the success of transfer of training, the results from this review suggest that fidelity is not a vital component to the overall success of transfer. It is important to note that fidelity may still potentially be an important factor in promoting transfer, however, no studies in this review had directly manipulated fidelity in training.

Although the level of fidelity may not be vital, fundamental differences in VEs compared to the real world could lead to negative transfer. In the case of Rauter et al.'s (2013) rowing study, participants in the VE training group showed an offset in their oar handling skills as compared to a real-world (on water) training group. In terms of biomechanical measures the VE training group degraded in 35%, stayed indifferent in 50% and improved in 15% (Rauter et al., 2013). In terms of a qualitative video analysis, the VE training group degraded in 21.4%, stayed indifferent in 53.5% and improved in 25% (Rauter et al., 2013). Perhaps if the training environment is too dissimilar to the real-world environment (e.g., lack of resistance while rowing in water) the training can lead to negative skill transfer. In the case of Todorov et al. (1997) study, the training task was uncharacteristic to the real-world task as participants had to focus on a computer screen while trying to connect with a ball and hit a target on a real table. In this study, negative transfer also occurred perhaps as participants performed a specific movement noticeably different to what occurs in the real world.

All the studies included in this review trained sports as closed skills, as conducted within a stable and predictable environment, unaffected by the presence of an interactive opponent or externally-paced condition. Tirp et al. (2015) studied transfer of dart throwing in a VE, which is a sport that utilizes closed skills by nature. However, the method that the remaining studies have used to study rowing (Rauter et al., 2013), baseball (Gray, 2017) and table tennis (Todorov et al., 1997) have also been assessed as closed skills, which is different to how they are played in real-world competition.

Due to the realism and flexibility of VEs, users can immerse in various game-like interactions among the presence of opponents and/or team members. Yet, to date, no studies have assessed the transfer of open skills to the real world (after training in a VE) to a control group. Wang et al. (2013) suggest that open skill sports require athletes to exhibit a higher level of concentration and speed in response, relative to athletes in closed skill sports. However, the value of VEs in open skill situations is unknown as sports which require interactive actions are likely to differ from static (closed skill) sports.

Streuber et al. (2012) tested this assumption as participants performed table tennis strokes in a VE while viewing an interactive virtual opponent and responding to their hits. Having the ability to see an opponent's body and their paddle was found to improve decision-making and preparation in their own stroke response (Streuber et al., 2012). This study was not included in the present review based on the absence of a measure of real-world sports performance. However, these findings suggest that there is value in VE-based training to train open skills, as it was found that people can improve by observing an opponent's movements and adapting to unpredictable responses in a VE (Streuber et al., 2012).

For the two studies which included participants with an intermediate level of experience, there is a rationale for training involving open skills. However, in consideration of the four studies in this review which used novices, there is basis for training involving closed skills. This is based on the consideration that learning the fundamentals (i.e., basic strokes and movements) in a competitive (open skill) environment might not be ideal for a novice. Future research is needed to investigate further if the open-closed factor in VE training is more so dependent upon the stage of learning of the player, and the skill set that is intended to be trained.

## Summary and Future Directions

In this review, we evaluated peer-reviewed research measuring the effectiveness of using VEs to improve real-world motor skills in sports. Collectively the evaluated studies support the notion that skills training in a VE can improve real-world sports performance. Notably, studies like Gray (2017) using complex adaptive training strategies show large real-world performance improvements thus demonstrating the potential value in VE based programs. VEs provide users with the flexibility to conveniently practice a wide range and number of skills, though this flexibility has not been entirely explored.

Few studies measured transfer and even fewer studies met the basic criteria for measuring real-world transfer. As a result, there was a lack of consistency regarding study design factors including the VE technology, length of the VE intervention, real-world performance measures and the presence or absence of a control group. As these factors were categorically different between studies it made comparisons and recommendations difficult. This limitation suggests that there is an immense need for such research, particularly for researchers considering VE training or companies developing VE-based training programs. Several companies' (such as STRIVR Willage, 2017, EON Sports, Beyond Sports, LucidCam and NeuroTrainer) market custom VEs and may make claims about the benefits of sports training in a VE, however, there is very little publicly available data supporting real-world improvements from VE sports training programs.

While there is some evidence that more basic closed skills are transferable from VE to the real-world, it remains to be established to what degree the current VE technology can be used to develop the more complex open skills. Researchers should consider introducing more competitive elements to training programs, such as opponents or teammates that match real world competitive environments. Including elements that mimic a real world competitive environment could further enhance open skills development. Some VE experiences aim to train open skills and incorporate an element of unpredictability, for example, interactions with an artificial intelligence opponent (Streuber et al., 2012). What is lacking, however, is controlled research studies that demonstrate transfer of open skills to the real-world.

Achieving an accurate in-game real-world performance assessment would be more challenging as the assessor/s would have less control over the events during gameplay. For example, in a football game with various opponents and teammates a player's performance is likely to vary to some degree on each occasion due to the interactions from both opponents and teammates. One approach that has been used successfully to analyse real-world performance was Gray (2017) whereby participants' league statistics and level of competition was assessed 5 years post VE training. Additional factors such as a player's in-game performance statistics and league rankings over a period of time can provide a more comprehensive overview though this can be both costly and time consuming.

There are a multitude of possibilities that may account for the limited number of studies measuring real-world transfer from a VE. Transfer is not a new term, but high quality VEs that have the capability to be used for sports training is still a relatively novel concept. One possibility is that research has not yet caught up with the influx of new virtual reality devices entering the consumer market. Another possibility is that assessing transfer of training has not been a research priority as some VEs have been developed as pure recreational games (e.g., Eleven: Table Tennis VR; developed by Fun Labs). Furthermore, some types of sports (e.g., water and interactive multiplayer sports) are costly and difficult to simulate with the current state of the technology. For a wider uptake of VE in sports training, it is important that real-world transfer is demonstrated. We have summarized important factors for measuring real-world transfer in Table 2. Researchers that may be interested in using VEs as a tool for training could incorporate these factors in their study design or build upon this research.

**Table 2:** Summary of factors to consider when assessing transfer of training from a virtual environment to the real world.

**Factor**	**Explanation**
Real-world assessment	An assessment of real-world performance on a task related to the one performed during VE training, both before and after training
Control group	A group which completes either no training or another form of VE training must be included to control for basic practice effects. A real-world training control group is needed if the goal is to compare the effectiveness of VE and real-world training. A no-training control group could be utilized to assess if VE training is greater than no training and to determine the minimal detectable change
Random allocation of participants	Random allocation of groups is essential to counter bias during the selection of the different groups
Blinding of assessor	The real-world assessor should remain blinded, meaning unknowing of which condition each participant has been assigned to limit bias during an assessment
Comprehensive assessment (additional)	In addition to pre- and post-assessments, other skills should be considered including performance under competitive conditions, such as in-game statistics for athletes involved in amateur/professional competitions

## Author Contributions

SM and AS: article selection and screening, writing, and analysis. TL: writing and analysis.

### Conflict of Interest

The authors declare that the research was conducted in the absence of any commercial or financial relationships that could be construed as a potential conflict of interest.
